# Mosses reduce soil nitrogen availability in a subarctic birch forest via effects on soil thermal regime and sequestration of deposited nitrogen

**DOI:** 10.1111/1365-2745.13567

**Published:** 2020-12-21

**Authors:** Marianne Koranda, Anders Michelsen

**Affiliations:** ^1^ Terrestrial Ecology Section Department of Biology University of Copenhagen Copenhagen Denmark; ^2^ Center for Permafrost University of Copenhagen Copenhagen Denmark; ^3^ Division of Terrestrial Ecosystem Research Centre for Microbiology and Environmental Systems Science University of Vienna Vienna Austria

**Keywords:** bryophytes, extracellular enzyme activities, microbial communities, microbial processes, moss, nitrogen availability, nitrogen cycling, soil temperature

## Abstract

1. In high‐latitude ecosystems bryophytes are important drivers of ecosystem functions. Alterations in abundance of mosses due to global change may thus strongly influence carbon (C) and nitrogen (N) cycling and hence cause feedback on climate. The effects of mosses on soil microbial activity are, however, still poorly understood. Our study aims at elucidating how and by which mechanisms bryophytes influence microbial decomposition processes of soil organic matter and thus soil nutrient availability.
2. We present results from a field experiment in a subarctic birch forest in northern Sweden, where we partly removed the moss cover and replaced it with an artificial soil cover for simulating moss effects on soil temperature and moisture. We combined this with a fertilization experiment with ^15^N‐labelled N for analysing the effects of moss N sequestration on soil processes.
3. Our results demonstrate the capacity of mosses to reduce soil N availability and retard N cycling. The comparison with artificial soil cover plots suggests that the effect of mosses on N cycling is linked to the thermal insulation capacity of mosses causing low average soil temperature in summer and strongly reduced soil temperature fluctuations, the latter also leading to a decreased frequency of freeze‐thaw events in autumn and spring. Our results also showed, however, that the negative temperature effect of mosses on soil microbial activity was in part compensated by stimulatory effects of the moss layer, possibly linked to leaching of labile substrates from the moss. Furthermore, our results revealed that bryophytes efficiently sequester added N from wet deposition and thus prevent effects of increased atmospheric N deposition on soil N availability and soil processes.
4. *Synthesis*. Our study emphasizes the important role of mosses in carbon and nutrient cycling in high‐latitude ecosystems and the potential strong impacts of reductions in moss abundance on microbial decomposition processes and nutrient availability in subarctic and boreal forests.

## Graphical abstract

We performed a moss removal/artificial soil cover experiment combined with a nitrogen fertilization experiment in a subarctic birch forest in order to investigate the mechanisms by which mosses influence microbial decomposition processes of soil organic matter and soil nutrient availability.

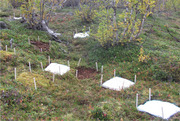

## 1 INTRODUCTION

In high‐latitude ecosystems bryophytes are strong drivers of ecosystem functions and constitute a major component in terms of biomass and productivity (Nilsson & Wardle, 2005; Oechel & Van Cleve, 1986; Street et al., 2013; Turetsky, 2003). The abundance of bryophytes, however, has been observed to decline as a result of global change (Jägerbrand et al., 2009; Jorgenson et al., 2015; Lang et al., 2012; Sorensen et al., 2012; Walker et al., 2006), which is linked to increasing vascular plant biomass and resulting enhanced shading (Alatalo et al., 2015; Jägerbrand et al., 2012; van der Wal et al., 2005; van Wijk et al., 2004), but may also be related to enhanced drought stress (Bragazza, 2008; Lang et al., 2012; Sorensen et al., 2012) or negative effects of increased nitrogen deposition on bryophyte physiology (Koranda et al., 2007; Pearce & van der Wal, 2002). Such shifts in vegetation composition of high‐latitude ecosystems may strongly influence soil microbial communities and thus impact nutrient availability and carbon storage, which may further cause feedback on climate change (Gundale et al., 2012; Wardle & Zackrisson, 2005; Wookey et al., 2009).

Bryophytes fundamentally differ from higher plants as they lack the ability to regulate water content and do not have roots nor a developed vascular system. Thus, they are also particular in the ways they influence soil processes and nutrient cycling. Mosses influence soil processes via four important functions: (a) Mosses strongly affect soil physical properties. Due to the low thermal conductivity of moss biomass, thick bryophyte layers have been shown to insulate the soil, causing lower soil temperature in summer and reduced soil temperature fluctuations (Gornall et al., 2007; Soudzilovskaia et al., 2013; Startsev et al., 2007). Mosses also tend to reduce soil evaporation, thus increasing soil moisture (Blok et al., 2011). (b) As bryophytes lack roots, they take up a large proportion of nutrients from atmospheric sources all over their surface, efficiently sequestering nutrients from atmospheric deposition, throughfall and litter leachates (Bates, 1992; Forsum et al., 2006; Turetsky, 2003). Moss layers hence function as a filter which retains absorbed nutrients for long time via internal recycling (Eckstein, 2000; Gundale et al., 2011; Rousk et al., 2014). On the other hand, many moss species are associated with N_2_‐fixing cyanobacteria which may provide important N input in high‐latitude ecosystems (DeLuca et al., 2002; Lindo et al., 2013; Rousk et al., 2013). (c) The absence of roots also implies the lack of root exudates. Pulses of labile (carbon) compounds may, however, be leached from bryophyte layers into the soil after drying–rewetting cycles and serve as energy source for soil microbes (Slate et al., 2019; Wilson & Coxson, 1999). (d) Decomposition rates of bryophyte litter are considerably lower than those of vascular plant litter (Hobbie, 1996; Lang et al., 2009), which has been ascribed to a high content of lignin‐like compounds in moss biomass, specific cell wall polysaccharides or cell components with antimicrobial properties (Hájek et al., 2011; Turetsky, 2003; Verhoeven & Toth, 1995).

Despite the important role of mosses in C and nutrient cycling of high‐latitude ecosystems, still little is known about the influence of bryophytes on soil microbial activity and microbial nutrient dynamics in soil. This study thus aims at elucidating how and by what mechanisms bryophytes influence microbial decomposition processes of soil organic matter (SOM), microbial community composition and hence soil nutrient availability. This also requires investigation of extracellular enzymes produced by soil microbes, which catalyse the first and often rate‐limiting step in decomposition of macromolecules (Burns et al., 2013; Wallenstein & Weintraub, 2008).

Effects of specific plant functional types on ecosystem processes have frequently been investigated using plant removal experiments (Bret‐Harte et al., 2004; Díaz et al., 2003; Wardle & Zackrisson, 2005). In such experiments, consisting of the removal of one or several plant functional types from a mixed plant community, the effect of a certain plant functional type on soil processes, nutrient cycling, etc., is, however, often difficult to estimate, as the remaining plants react to the removal of other plant functional groups. In this study we thus (largely) excluded vascular plants for investigating moss effects on soil processes.

We performed a field experiment in a subarctic birch forest in northern Sweden, where we partly removed the moss cover and replaced it with an artificial soil cover. The artificial soil cover should simulate moss effects on soil temperature and moisture while lacking other properties of mosses like N uptake capacity and release of labile substrates. We combined this experiment with a fertilization experiment with ^15^N‐labelled N for analysing the effects of moss N sequestration on soil processes. We hypothesized (a) that microbial biomass would be lower and N‐cycling slower in moss plots compared to moss removal plots and (b) that a manipulation of soil physical properties by an artificial soil cover would affect microbial biomass and nutrient cycling similar to a moss cover. Furthermore, we expected (c) that N fertilization would not affect soil microbial processes in moss plots as the added N would be sequestered by the moss layer, while N fertilization would increase microbial biomass and alter the pattern of extracellular enzyme activities in the moss removal plots both with and without artificial soil cover.

## 2 MATERIALS AND METHODS

### 2.1 Site description

We established the field experiment near Abisko in northern Sweden in early September 2016. Average air temperature recorded at the nearby research station was 12°C in July and −10°C in January, with an annual precipitation of 376 mm. Vegetation at the study site was an open birch forest (*Betula pubescens*) with an understorey of a continuous moss cover (feathermosses, *Hylocomium splendens* and *Pleurozium schreberi*) and scattered ericaceous dwarf shrubs (mainly *Empetrum hermaphroditum, Vaccinium vitis‐idaea, Vaccinium myrtillus*). Soil type was a gleyic Podzol, consisting of a peaty organic horizon (3–10 cm depth) underlain by a shallow mineral horizon, partly with a light eluvial horizon. The organic soil was characterized by ~37% total C, ~1.35% total N and a mean organic matter content of 84%. The pH‐value of the organic horizon was between 4.2 and 4.3.

### 2.2 Experimental setup

The experiment was established in a fully crossed design of four soil cover types (moss, bare soil, foam material and fleece) and two fertilization treatments (fertilized and non‐fertilized), replicated in six blocks within an area of 2,000 m^2^. The plots were 50 cm × 50 cm in size and were located between 20 cm and 2 m apart, depending on the blocks (Figure S1). Care was taken in the selection of the plots that no downhill leaching of fertilizer from fertilized to non‐fertilized plots could occur. The borders of all plots were cut using a spade to interrupt root ingrowth.

In the moss plots the moss cover was left intact, only scattered dwarf shrubs were removed by gently pulling them out of the soil, if easily possible, or clipping. Moss carpets were, however, carefully lifted and put back in place, so that disturbance in all treatments was comparable. As the moss layers could easily be separated from underlying peaty soil, lifting could be done without causing destruction. In the bare soil and artificial soil cover plots all vegetation, including mosses (live green and dead brown moss) and vascular plants, was removed. The artificial soil cover plots were then covered with either foam material consisting of open cell polyurethane (two layers of strips of 10–15 cm width and 2 cm thickness), or polyester fleece (material used as e.g. filling in blankets, also two layers of 2 cm thickness). We had chosen those materials because of their water holding capacity (Table S1) and their inertness, which had been tested in a pre‐experiment. The soil cover was fixed with a coarse meshed net and hooks.

The N‐fertilized plots (one plot per soil cover treatment per block) received NH_4_NO_3_ (containing 10% ^15^N) at a total N load of 1.5 g N m^−2^ year^−1^, which was applied in two doses of 0.75 g N/m^2^ in mid‐September 2016, after setting up the plots, and in early June 2017, right after snow melt. This N load accounts for about 10 times the atmospheric background N deposition (~0.15 g N m^−2^ year^−1^; Phil‐Karlsson et al., 2009). The fertilizer was applied dissolved in water (300 ml per plot, equivalent to 1.2 mm rainfall), by means of a bottle with fine holes in the lid. The non‐fertilized plots received an equal volume of water as the fertilized plots.

### 2.3 Soil temperature and moisture

Soil temperature at 3 cm depth (measured from the top of the organic horizon) was recorded every 30 min throughout a year in three replicate plots per soil cover treatment by Tinytag temperature loggers (TGP‐4020 with PB‐5015‐1M5 sensors). Additionally, manual temperature measurements were performed every second week between end of June and end of July 2017 in all 48 plots (12 replicates per soil cover treatment) using thermometers. Measurements were taken in the afternoon (2 p.m.–4 p.m.), thus the values represent approximate daily maximum soil temperatures. Furthermore, average summer soil temperature in all plots was determined with Ambrose thermal cells (TH cells, Woden, Australia). The cells consist of polycarbonate resin capsules embedded in water‐filled cells, which acquire moisture at a temperature dependent rate and thus yield an integrated temperature over the incubation period (Jonasson et al., 1993).

The number of freeze‐thaw cycles per year was calculated from the number of zero transitions of the continuous temperature measurements, divided by two. As soil temperature sometimes oscillated closely around zero, we performed a second calculation only counting freezing and thawing events, when soil temperature exceeded 0 ± 0.1°C.

Soil moisture at 0–6 cm depth was measured every second week in all plots between end of June and end of July 2017 using a Theta‐probe (ML2, Delta‐T Devices Ltd). At each time point three measurements per plot were performed and averaged.

### 2.4 Soil sampling

Soil sampling was performed on 10 July 2017. Five to ten soil cores of 4 cm diameter were taken of the entire depth of the organic horizon (3–10 cm) in each plot and bulked. The number of soil cores taken per plot varied depending on the horizon depth, i.e. in case of shallow organic horizon more soil cores were taken in order to collect a sufficient amount of soil. Furthermore, pieces of moss cushions (comprising live green and dead brown moss) and foam/fleece material (5 × 5 cm) were collected from the moss and artificial soil cover plots.

Roots were removed from the soil samples, and soil samples were homogenized by hand. Samples were stored at 4°C until further processing. Soil extractions were performed on the following day and all other assays within 8 days after soil sampling.

### 2.5 Total C and N and recovery of fertilizer N

Subsamples of soil and moss/soil cover were oven‐dried (at 90°C and 60°C, respectively), ground in a ball mill and analysed for total C and N and δ^15^N by an Eurovector elemental analyser coupled to an Isoprime IRMS. Samples were analysed with reference gas calibrated against international working standards IAEA N1, N2 and USGS 25, 26, 32 and drift corrected using internal standards of leaf (peach, NIST 1547) material calibrated with these standards. As grinding was not possible for the foam material and fleece, thin columns were cut out of the material (through the entire layer) and packed into tin capsules for IRMS analysis. Recovery of fertilizer N in moss/soil cover and soil was calculated according to the following equations:

$$R=\left(\left[{}^{15}N_{\text{soil/cover}}\right]\;\times \;{\text{mass}}_{\text{soil/cover}}\right)/{}^{15}N_{\text{applied}}\;\times \;100,$$

where *R* is the recovery of fertilizer N in percent, [^15^N_soil/cover_] is the concentration of ^15^N in soil and cover/moss (in mg/g DW), mass_soil/cover_ is the total dry weight of organic soil and cover/moss in g per plot and ^15^N_applied_ is the amount of ^15^N applied in mg per plot.

$$\left[{}^{15}N_{\text{soil/cover}}\right]=\left({\%}^{15}N_{\text{soil/cover}}‐{\%}^{15}N_{\text{background}}\right)\;\times \;N/10,$$

where [^15^N_soil/cover_] is the concentration of ^15^N in soil and cover/moss (in mg/g DW), %^15^N_soil/cover_ is atom %^15^N in soil and cover/moss of fertilized plots, %^15^N_background_ is atom %^15^N in soil and cover/moss of non‐fertilized plots and N is the N content of soil and cover/moss (in percent of DW). Atom %^15^N was calculated from δ^15^N values using the equation:

$$\begin{array}{cc} \text{atom}\;{\%}^{15}N & =100\;\times \;\text{Std}\;\times \;\left(\delta^{15}N_{\text{sample}}/1000+1\right)/\\ & \;\;\;\left(1\;+\;\text{Std}\;\times \;\left(\delta^{15}N_{\text{sample}}/1000\;+\;1\right)\right), \end{array}$$

where Std is the fractional abundance of ^15^N in the isotopic standard (N_2_ in air).

### 2.6 C and N pools

Subsamples of fresh soil (7.5 g) were extracted with 50 ml of water for 1 hr and vacuum‐filtered through glass fibre filters (Whatman GF/D). Concentrations of ${\text{NH}}_{4}^{+}$ and ${\text{NO}}_{3}^{‐}$ were determined by flow‐injection analyser (Fiastar 5000, FOSS analytical), using applications AN 5220 for ${\text{NH}}_{4}^{+}$ and AN5201 for ${\text{NO}}_{3}^{‐}$ respectively. Concentrations of dissolved organic C and total dissolved N were analysed with a TOC/TN analyser (Shimadzu). Dissolved organic N (DON) concentration was calculated from the difference of total dissolved N and inorganic N.

δ^15^N of dissolved N was determined by freeze‐drying of water extracts and subsequent wiping of dried residues using quartz filters, which were then subjected to IRMS analysis (Ravn et al., 2017). The concentration of dissolved N originating from fertilizer N and from native soil N, respectively, was then calculated according the following equations:

$$\left[{\text{TDN}}_{\text{fert}}\right]=\left[\text{TDN}\right]\;\times \;{\text{APE}}^{15}N_{\text{TDN}}\;\times \;10,$$

where [TDN_fert_] is the concentration of total dissolved N originating from fertilizer (in mg/g OM), [TDN] is the concentration of total dissolved N in water extracts (in mg/g OM) and APE ^15^N_TDN_ is atom percent excess ^15^N of total dissolved N (atom %^15^N_sample_ – atom %^15^N_background_)

$$\left[{\text{TDN}}_{\text{native}}\right]=\left[\text{TDN}\right]‐\left[{\text{TDN}}_{\text{fert}}\right],$$

where [TDN_native_] is the concentration of total dissolved N originating from native soil N.

Microbial biomass was determined by the fumigation‐extraction method (Brookes et al., 1985). An extraction coefficient of 0.45 for C (Wu et al., 1990) and 0.4 for N (Jonasson et al., 1996) was used to account for incomplete extraction of microbial biomass C and N.

### 2.7 Microbial community structure

The abundance of microbial groups was estimated from phospholipid fatty acids (PLFAs) using a modified method after Buyer and Sasser (2012). After extraction of freeze‐dried soil samples by a mixture of methanol, chloroform and citrate buffer (2:1:0.8, v/v/v), PLFAs were separated from neutral lipids on silica columns and subjected to alkaline methanolysis. Dried fatty acid methyl esters were re‐dissolved in isooctane and concentrations of PLFAs were determined on a gas chromatograph (Trace GC Ultra, Thermo Scientific) equipped with a DB‐23 column. A mixture of fatty acid methyl esters (FAMEs; Supelco, nr. 47080‐U and 47885‐U) was used as a qualitative standard. An internal standard (19:0) was used for calculation of FAME concentrations. We used the sum of the fatty acids i15:0, a15:0, i16:0, i17:0, a17:0, 17:0, 16:1ω9, 16:1ω7, cy17:0, cy19:0 as a measure for bacterial biomass and the quantity of 18:2ω6,9 as an indicator of fungal biomass.

### 2.8 Extracellular enzyme activities

Potential hydrolytic enzyme activities were estimated by microplate assays using fluorescent substrates (Bell et al., 2013; German et al., 2011). One gram of fresh soil was suspended in 100 ml of Na‐acetate buffer (pH 4.4) and mixed for 1 min using a kitchen blender. 200 µl of the soil suspensions were pipetted into black microtiterplates (three analytical replicates) and 50 µl of substrate solutions added: 1 mM 4‐MUF‐N‐acetyl‐β‐D‐glucosaminide was used for determination of chitinase activity, 2 mM 4‐MUF‐phosphate for phosphatase and 1 mM 4‐MUF‐β‐D‐cellobioside for cellobiosidase. Standard curves were prepared from 4‐methylumbelliferone (MUF) in six concentrations added to soil suspensions. Substrate blanks (substrate plus buffer, four replicates) were also included. Microplates were incubated at 12°C for 3 hr (phosphatase)–4 hr (chitinase, cellobiosidase), then fluorescence was measured using a spectrometer (Spectramax) with 365 nm excitation and 450 nm emission.

Potential peroxidase activity was measured photometrically (Saiya‐Cork et al., 2002). One millilitre of the soil suspensions was pipetted into 2 ml reaction tubes (four tubes per sample). One tube per soil sample was amended with 1 ml of 10 mM L‐3,4‐dihydroxyphenylalanine (DOPA), one tube received 900 µl of DOPA solution + 100 µl 0.3% H_2_O_2_. The remaining two tubes received buffer instead of DOPA solution (soil blanks). Substrate blanks were also prepared. After incubation at 12°C for 4 hr, samples were centrifuged, 250 µl of the supernatant transferred to clear microplates and absorbance at 460 nm was read on a spectrometer. Peroxidase activity was calculated from the difference in absorbance of samples with and without peroxide addition, divided by the extinction coefficient (7.9; Bach et al., 2013).

### 2.9 Gross N mineralization and consumption

Gross N mineralization rates were assessed using the ^15^N pool dilution technique (Kaiser et al., 2005; Myrold & Tiedje, 1986). ^15^NH_4_Cl was applied to two subsamples of fresh soil (2 g), then samples were incubated at 12°C for 4 and 24 hr respectively. A set of samples was not amended with ^15^N and was used for determination of background ^15^N values. After extraction of samples with 2M KCl, ${\text{NH}}_{4}^{+}$ was diffused into acid traps, which were dried and analysed by an Eurovector elemental analyser coupled to an Isoprime IRMS. Gross N mineralization rates and gross ${\text{NH}}_{4}^{+}$ consumption rates (i.e. the sum of all ${\text{NH}}_{4}^{+}$ consuming processes including microbial immobilization and nitrification) were calculated according to the following equations:

$$\text{grossmin}\;=\;\left(A_{t}‐A_{0}\right)/t\;\times \;\left(\ln\left({\text{APE}}_{0}/{\text{APE}}_{t}\right)/\ln\left(A_{t}/A_{0}\right)\right),$$

$$\text{netmin}\;=\;(A_{t}‐A_{0})/t,$$

grosscons=grossmin‐netmin,

where grossmin is gross N mineralization, netmin is net N mineralization, grosscons is gross ${\text{NH}}_{4}^{+}$ consumption. *A_t_* is the ${\text{NH}}_{4}^{+}$‐N pool after time *t*, *A*_0_ is the initial ${\text{NH}}_{4}^{+}$‐N pool, APE (atom percent excess) is atom %^15^N‐${\text{NH}}_{4\;\text{sample}}^{+}$ – atom %^15^N‐${\text{NH}}_{4\;\text{background}}^{+}$.

### 2.10 Data analyses

The effect of soil cover type on average soil temperature and daily temperature amplitude in the continuously measured plots was estimated by repeated‐measures ANOVA of monthly averages, with cover type and month as fixed factors, and autocorrelation modelled as first‐order autoregressive (AR1). Separate analyses were conducted with data of the summer months (June–August) and autumn/early winter (September–November), i.e. the period before the establishment of a continuous snow cover. The effect of cover type on the fully replicated soil temperature and moisture data in summer and the recovery of fertilizer N was analysed by linear mixed effect models, with cover type as fixed factor and block as random factor, followed by Tukey's post‐hoc tests. Manual soil temperature and moisture data were averages of three time points. Data of C and N pools, abundance of microbial groups and microbial process rates were analysed using linear mixed effect model ANOVA with cover type and fertilization as fixed factors and block as random factor. The contribution of the random effect to the explained variance of the models was estimated by calculating marginal *R* ^2^ (fixed effects only) and conditional *R* ^2^ (fixed and random effects; Nakagawa & Schielzeth, 2013). Significant differences between treatments were estimated by Tukey's post‐hoc tests. Correlations between C and N pools, microbial groups and process rates were estimated by Spearman's correlation coefficients. The relationship between enzyme activities and soil physical parameters was analysed by linear regression models. Regression analyses were run with treatment averages, in order to take account of the non‐independence of data from the same treatment. Data of C and N pools, microbial groups and process rates were log‐transformed prior to analyses to meet the assumptions of normality and homogeneity of variances for linear models. All analyses were performed using R version 3.5.1 (R Core Team, 2018), with the packages nlme (Pinheiro et al., 2018), ‘lmerTest’ (Kuznetsova et al., 2017) and ‘MuMIn’ (Bartón, 2016).

## 3 RESULTS

### 3.1 Manipulation of soil physical parameters and N availability by the experimental treatments

**FIGURE 1:**
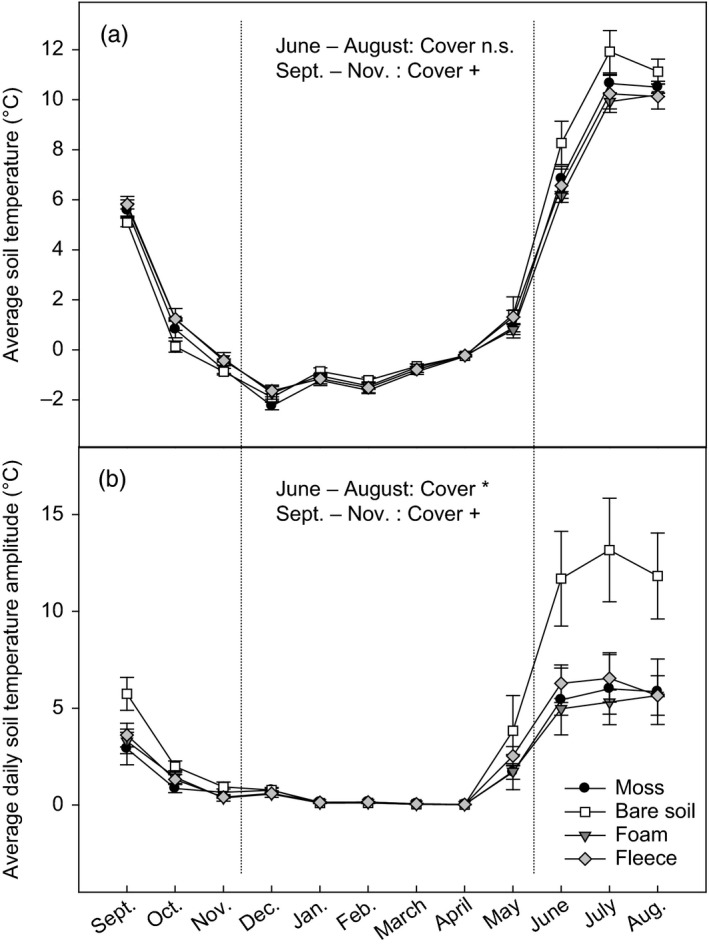
(a) Average soil temperature (at 3 cm depth) and (b) average daily soil temperature amplitude in moss plots, bare soil plots and plots covered with foam material and fleece respectively. Values are means ± *SE* (*n* = 3). Effects of soil cover determined by repeated‐measures ANOVA for summer months (June–August) and autumn/early winter (September–November) are indicated by *(*p* < 0.05), ^+^(*p* < 0.1) and n.s. (non‐significant)

The soil cover treatments affected the soil thermal regime throughout the snow‐free season in the subset of continuously measured plots: In autumn and early winter average soil temperature tended to be lower in the bare soil plots than in the fleece‐covered plots (Figure 1a), while in summer the bare soil plots exhibited on average 1.1°C higher soil temperature than the moss plots and 1.6°C higher soil temperature than the artificial soil cover plots, although these differences were not statistically significant (*F*_3,21_ = 1.64, *p* = 0.21; Figure 1a). The daily temperature amplitude, however, was twice as high in bare soil plots as in the moss plots and the artificial soil cover plots during the summer months (Cover effect: *F*_3,21_ = 3.43, *p* = 0.04; Figure 1b). The fully replicated soil temperature measurements performed in summer corroborated these results, showing a marginally significant effect of cover type on average summer soil temperature determined by the thermal cells (*F*_3,39_ = 2.20, *p* = 0.10; Table 1) and a highly significant cover effect on afternoon soil temperature (representing approximate daily maximum temperature; *F*_3,39_ = 32.10, *p* < 0.001; Table 1). The aimed simulation of moss effects on soil physical properties by means of an artificial soil cover was thus successful with respect to the soil temperature effects. This was, however, not the case for the soil moisture, which was slightly higher in the moss plots than in the bare soil plots and the fleece plots (post‐hoc test: *p* < 0.05, Table 1) and intermediate in the foam plots.

**TABLE 1:** Average soil temperature, afternoon soil temperature (at 2 p.m.–4 p.m.) and soil moisture in July (at 3 cm depth) in moss plots, bare soil plots and plots covered with foam material and fleece respectively. Recovery of fertilizer N in moss/soil cover and soil of plots fertilized with ^15^N‐labelled NH_4_NO_3_. Values are means (*SE* in parentheses), *n* = 12 (soil temperature and moisture data), *n* = 6 (recovery of fertilizer N). Significant differences between soil cover treatments determined by Tukey's post‐hoc tests are indicated by different letters (*p* < 0.05)

	Moss	Bare soil	Foam	Fleece
Average soil temperature in summer (°C)	8.7 (0.1)	9.1 (0.2)	8.7 (0.2)	8.9 (0.1)
Average afternoon soil temperature (July; °C)	11.6^c^ (0.2)	14.5^a^ (0.3)	11.9^c^ (0.3)	13.0^b^ (0.3)
Average soil moisture (July; %)	64^a^ (2)	56^b^ (3)	60^ab^ (2)	56^b^ (2)
Recovery of fertilizer N in moss/soil cover (% of added N)	89^a^ (16)	—	0.5^b^ (0.1)	0.2^c^ (0.0)
Recovery of fertilizer N in soil (% of added N)	4^c^ (1)	51^a^ (6)	18^b^ (6)	24^ab^ (6)

The moss removal also significantly altered the frequency of freeze‐thaw events. In the bare soil plots the number of freeze‐thaw cycles was more than four times as high as in moss plots and nearly three times as high as in fleece plots, while the foam‐covered plots showed intermediate values (Table S2). If a threshold of 0 ± 0.1°C was applied in the calculation of freeze–thaw cycles, the differences between the treatments were still greater.

Ten months after the first dose of N fertilization and 5 weeks after the second dose, 89% of the applied N was still in the moss layer (Table 1), while hardly any fertilizer N was recovered in the artificial soil cover. Only 4% of the added N was recovered in soil below the mosses, compared to 51% in the bare soil plots. In the artificial soil cover plots, recovery of fertilizer in soil was lower than in the bare soil (<25% of the added N) but five to six times higher than in the moss plots, meaning that the applied fertilizer had penetrated the foam and fleece material and entered the soil.

### 3.2 Effects of the experimental treatments on soil C and N pools and microbial processes

**FIGURE 2:**
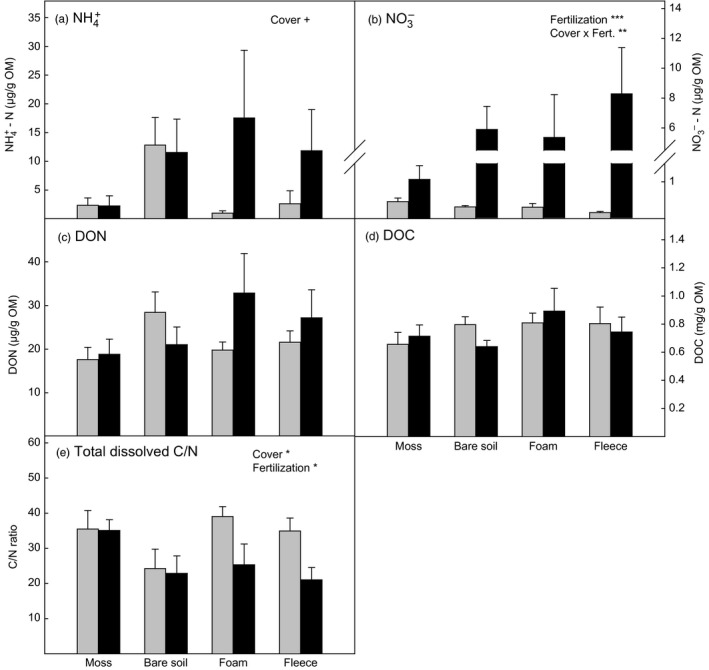
Concentration of (a) ammonium, (b) nitrate, (c) dissolved organic N, (d) dissolved organic C, (e) ratio of dissolved organic C to total dissolved N in water extracts of soil collected in moss plots, bare soil plots and plots covered with foam material and fleece respectively. Grey bars represent non‐fertilized plots and black bars represent plots fertilized with NH_4_NO_3_. Values are means ± *SE* (*n* = 6). Significant effects of cover type, fertilization and the interaction determined by linear mixed effect models are indicated by ***(*p* < 0.001), **(*p* < 0.01), *(*p* < 0.05) and ^+^(*p* < 0.1). Details on ANOVA models are presented in Table 2

The removal of the moss layer caused a more than five‐fold increase in the average concentration of ammonium in bare soil plots compared to the moss plots (Tukey's post‐hoc test: *p* = 0.07; Figure 2a) and a decline in the ratio of dissolved C to N by one third (post‐hoc test: *p* = 0.03, Figure 2e). In the non‐fertilized plots moss removal tended to enhance the availability of both ammonium and dissolved organic N (DON; Figure 2a,c). This effect was, however, not observed, if the moss layer was replaced by an artificial soil cover. N fertilization strongly amplified the availability of nitrate, the least abundant form of N, in bare soil plots and artificial soil cover plots (Figure 2b; Table 2), while the fertilization effect on the concentrations of ${\text{NH}}_{4}^{+}$ and DON was less pronounced in the artificial soil cover plots and was not existent or even tended to be negative in the bare soil plots. The ratio of dissolved C to N was reduced by more than 40% by the fertilization in the artificial soil cover plots, leading to values similar to the bare soil plots (Figure 2e). In the moss plots, however, soil N availability was not affected by the fertilization treatment.

**TABLE 2:** Results of linear mixed effect model ANOVA estimating effects of soil cover treatments and N fertilization on C and N pools, microbial biomass, abundance of microbial groups and microbial processes. *F*‐values for main effects and interaction, significant values are in bold (***p < 0.001, **p < 0.01, *p < 0.05, ^+^ *p* < 0.1), marginal *R* ^2^ ($R_{\left(m\right)}^{2}$, fixed effects only) and conditional *R* ^2^ ($R_{\left(c\right)}^{2}$, fixed and random effects)

	Cover (*df* = 3)	Fertilization (*df* = 1)	Cover × fert. (*df* = 3)	$R_{\left(m\right)}^{2}$	$R_{\left(c\right)}^{2}$
${\text{NH}}_{4}^{+}$	**2.37^+^**	2.76	1.58	0.20	0.36
${\text{NO}}_{3}^{‐}$	1.74	77.61***	5.50**	0.68	0.68
DON	1.34	0.17	1.59	0.13	0.30
DOC	1.78	0.39	1.20	0.11	0.44
Dissolved C/N	3.15*	6.44*	1.79	0.27	0.40
Microbial biomass C	0.43	0.05	0.99	0.07	0.21
Microbial biomass N	0.32	0.28	0.87	0.07	0.13
Microbial biomass C/N	0.27	0.69	0.47	0.05	0.25
Total PLFAs	6.08**	0.09	2.07	0.40	0.50
Bacterial PLFAs	6.74**	0.88	2.15	0.43	0.52
Fungal PLFAs	0.08	0.70	0.36	0.05	0.24
Chitinase	5.80**	0.26	0.52	0.20	0.51
Phosphatase	3.88*	0.31	1.94	0.24	0.35
Chit./phos.	6.64**	0.12	1.38	0.23	0.56
Cellobiosidase	4.83*	0.60	1.56	0.18	0.56
Peroxidase	2.13	0.01	0.05	0.12	0.17
Perox./cellobios.	**2.24^+^**	0.10	0.24	0.13	0.20
Gross N mineralization	1.20	0.03	0.21	0.08	0.11
Gross ${\text{NH}}_{4}^{+}$ consumption	1.25	0.00	0.50	0.10	0.10

Microbial biomass was not significantly changed by either the soil cover type or the N fertilization (Tables 2 and 3), but showed a positive correlation with the availability of DON (*r* = 0.56, *p* < 0.001) and ammonium (*r* = 0.43, *p* < 0.01). The soil cover treatment did, however, influence the microbial community structure (Tables 2 and 3). The abundance of bacterial PLFAs and of total PLFAs was highest in non‐fertilized bare soil plots and lowest in the plots with foam material, while no differences in fungal abundance among the cover types were observed. Interestingly, these differences among the cover types were not apparent in the fertilized plots. Like microbial biomass N, bacterial abundance was positively correlated with N availability (*r* = 0.58, *p* < 0.001 and *r* = 0.49, *p* < 0.01 for DON and ${\text{NH}}_{4}^{+}$ respectively).

**TABLE 3:** Microbial biomass C and N and microbial community composition estimated by abundance of bacterial and fungal PLFAs in soil collected in moss plots, bare soil plots and plots covered with foam material and fleece respectively. Plots were either fertilized with NH_4_NO_3_ or non‐fertilized. Values are means (*SE* in parentheses), *n* = 6 (microbial biomass), *n* = 4 (PLFAs). ANOVA results are presented in Table 2

	Moss		Bare soil		Foam material		Fleece	
	Non‐fert.	Fertilized	Non‐fert.	Fertilized	Non‐fert.	Fertilized	Non‐fert.	Fertilized
Microbial biomass C (mg/g OM)	8.0 (0.4)	8.0 (0.3)	8.5 (0.6)	7.7 (0.8)	7.3 (0.2)	7.8 (0.4)	7.8 (0.2)	7.8 (0.4)
Microbial biomass N (mg/g OM)	1.38 (0.13)	1.33 (0.12)	1.51 (0.13)	1.37 (0.12)	1.19 (0.07)	1.46 (0.17)	1.32 (0.13)	1.44 (0.09)
Microbial biomass C/N ratio	6.0 (0.6)	6.2 (0.5)	5.8 (0.5)	5.6 (0.3)	6.2 (0.4)	5.8 (0.7)	6.3 (0.8)	5.5 (0.2)
Total PLFAs (nmol/g OM)	979 (64)	817 (104)	1,149 (137)	940 (39)	635 (83)	761 (85)	785 (58)	876 (65)
Bacterial PLFAs (nmol/g OM)	338 (36)	264 (52)	412 (40)	317 (46)	193 (20)	244 (38)	286 (22)	297 (17)
Fungal PLFAs (nmol/g OM)	160 (38)	151 (41)	177 (76)	175 (27)	148 (28)	158 (22)	124 (32)	163 (25)
Fungal/bacterial PLFAs	0.51 (0.15)	0.66 (0.19)	0.46 (0.23)	0.61 (0.14)	0.75 (0.09)	0.73 (0.22)	0.45 (0.13)	0.55 (0.07)

**FIGURE 3:**
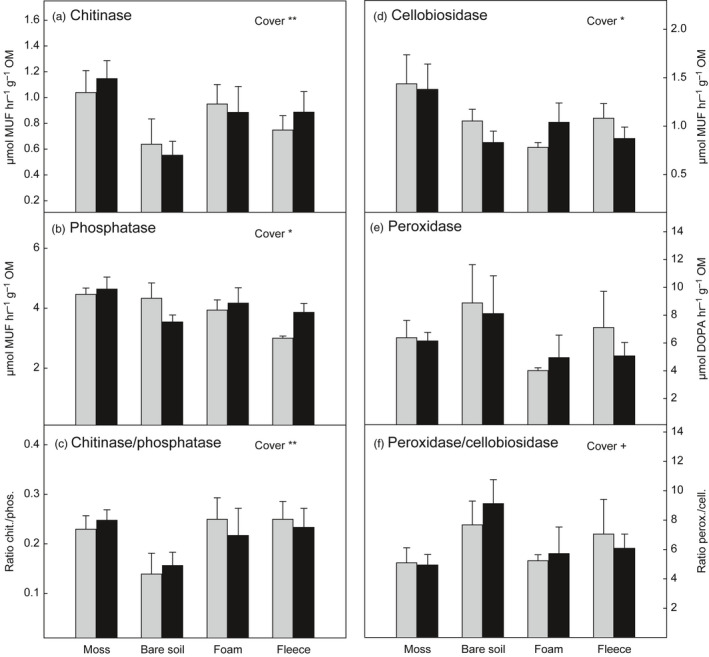
Potential extracellular enzyme activities measured in soil collected in moss plots, bare soil plots and plots covered with foam material and fleece respectively. Grey bars represent non‐fertilized plots and black bars represent plots fertilized with NH_4_NO_3_. (a) chitinase (N‐acetylglucosaminidase), (b) phosphatase, (c) ratio of chitinase to phosphatase, (d) cellobiosidase, (e) peroxidase, (f) ratio of peroxidase to cellobiosidase. Values are means ± *SE* (*n* = 6). Significant effects of cover type determined by linear mixed effect models are indicated by **(*p* < 0.01), *(*p* < 0.05) and ^+^(*p* < 0.1). Effects of fertilization and the interaction were non‐significant for all enzyme data. Details on ANOVA models are presented in Table 2

In contrast to the N pools, the activities of extracellular enzymes were clearly affected by the type of soil cover, but showed surprisingly little changes in response to N addition (Figure 3; Table 2). Chitinase activity was around 45% lower in the bare soil plots than in the moss plots (post‐hoc test: *p* < 0.01; Figure 3a). Similarly, the ratio of chitinase to phosphatase (as a measure of N‐degrading vs. P‐degrading activities) was significantly reduced by the moss removal (post‐hoc test: *p* < 0.01; Figure 3c), but not if the plots were covered with foam or fleece material. The activities of hydrolytic enzymes (chitinase, phosphatase and cellobiosidase) were generally highest in the moss plots, while peroxidase activity was highest in the bare soil plots (Figure 3a–e). The ratio of peroxidase to cellobiosidase activity (as a measure of oxidative vs. cellulolytic activities) tended to be higher in bare soil plots than in the moss and artificial soil cover plots (Figure 3f). Regression analysis of enzyme activities and soil physical parameters revealed a significant negative dependence of chitinase activity on mean summer soil temperature and afternoon soil temperature, respectively (*r* ^2^ = 0.48, *p* < 0.1 and *r* ^2^ = 0.79, *p* < 0.01, Table S3), and a positive dependence of peroxidase activity on soil temperature (*r* ^2^ = 0.72, *p* < 0.01 and *r* ^2^ = 0.60, *p* < 0.05 for mean and afternoon soil temperature respectively). Chitinase and phosphatase activity were positively related with soil moisture (*r* ^2^ = 0.72, *p* < 0.01 and *r* ^2^ = 0.53, *p* < 0.05 respectively).

**FIGURE 4:**
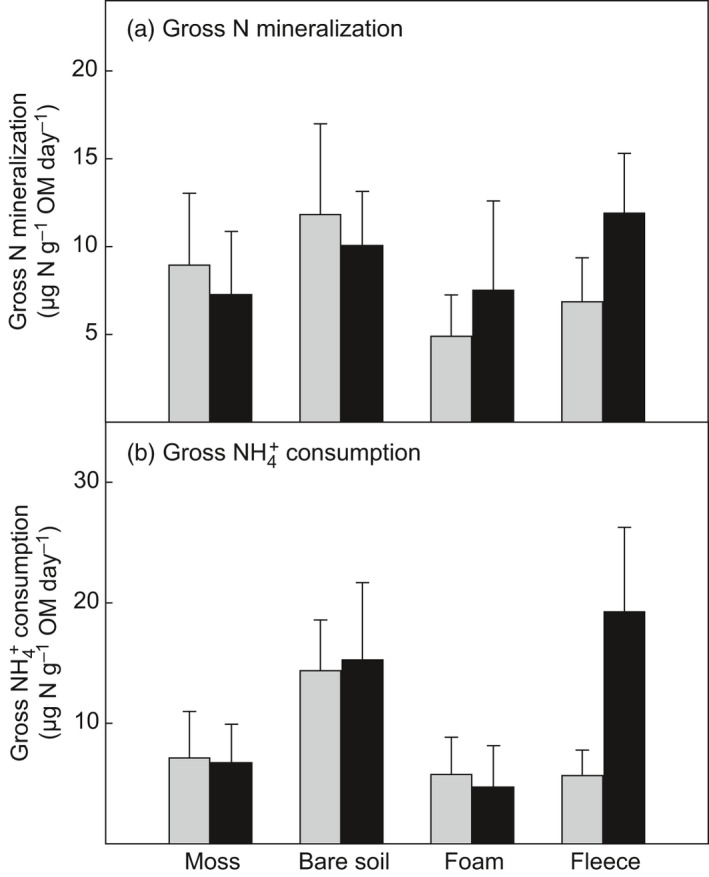
(a) Gross N mineralization (ammonification) rate and (b) gross ${\text{NH}}_{4}^{+}$ consumption rate measured in soil collected in moss plots, bare soil plots and plots covered with foam material and fleece respectively. Grey bars represent non‐fertilized plots and black bars represent plots fertilized with NH_4_NO_3._ Values are means ± *SE* (*n* = 6). ANOVA results are presented in Table 2

Gross N mineralization rates and gross ${\text{NH}}_{4}^{+}$ consumption rates were both highest in the bare soil plots (Figure 4), but the effect of cover type was not significant (Table 2). Gross N mineralization rates correlated with the availability of ${\text{NH}}_{4}^{+}$ (*r* = 0.51, *p* < 0.001) and DON (*r* = 0.44, *p* < 0.01).

## 4 DISCUSSION

Bryophytes play a crucial role in carbon and nutrient cycling of high‐latitude ecosystems. This study aimed at elucidating the mechanisms by which bryophytes influence microbial decomposition processes of SOM and nitrogen cycling in a subarctic birch forest.

As demonstrated previously in several studies, mosses exert strong influence on the soil thermal and moisture regime. Results from tundra heath and boreal forests revealed that thick moss layers reduce average soil temperature in summer (Gornall et al., 2007; Startsev et al., 2007; Street et al., 2018) and decrease the daily fluctuations in soil temperature during the growing season (Gornall et al., 2007; Soudzilovskaia et al., 2013), because of the low thermal conductivity of moss biomass (Blok et al., 2011; Soudzilovskaia et al., 2013). In our study the increase in mean summer soil temperature in the bare soil plots compared to the moss plots was not statistically significant (Figure 1; Table 1), while the daily soil temperature amplitude was strongly enhanced. A previous study from the same area investigating a range of moss species (Soudzilovskaia et al., 2013) also found pronounced effects of mosses on soil temperature amplitude, but not on average growing season soil temperature. Some divergence in the reported results on moss effects on soil thermal regime may be linked to the precipitation regime at the study sites. As moss removal increases evapotranspiration (Blok et al., 2011), enhanced evaporative cooling may partly compensate for the warming effect of the moss removal, which was most likely also the case in our study.

In our experiment we used two types of artificial soil cover in order to simulate moss effects on soil temperature and moisture. This should allow us to elucidate how far the effects of bryophytes on soil processes are due to moss effects on soil microclimate and to disentangle this from other effects of the moss layers. The artificial soil cover generally affected the soil thermal regime similar to the moss layer (Figure 1; Table 1). Afternoon soil temperature in the plots covered with fleece was, however, slightly higher than in the plots with foam material and the moss plots (Table 1), and soil moisture was lower in the fleece than in the moss plots (Table 1), suggesting higher thermal conductivity and higher evapotranspiration through the fleece than the foam material.

The manipulations of soil physical properties by the moss removal and artificial soil cover treatments translated into changes in N availability, as well as altered enzyme activities and N‐cycling processes. The bare soil plots showed increased availability of dissolved N compared to the moss plots and the non‐fertilized plots with artificial soil cover (Figure 2). This is in line with results from Gornall et al. (2007) and Bret‐Harte et al. (2004), who reported enhanced levels of inorganic N in soil as a result of experimentally thinned moss layers or moss removal in tundra ecosystems. Another study, however, did not find an effect of moss removal on soil N availability in a boreal forest (Startsev et al., 2007), which could be due to lateral translocation of N and uptake by mycorrhiza and roots. In our study, lateral transport of N out of the plots and plant N uptake was prevented by the trenching around the plots. The high N availability in the (non‐fertilized) bare soil plots corresponded with high bacterial abundance, microbial biomass N (Table 3), as well as high gross N mineralization rates and ${\text{NH}}_{4}^{+}$ consumption rates (Figure 4), which means that the moss removal promoted the establishment of a bacterial dominated microbial community characterized by rapid N‐turnover, in line with our first hypothesis.

The high N availability in the bare soil plots was also mirrored by the pattern of extracellular enzyme activities. As soil microbes regulate the production of extracellular enzymes to meet their C and nutrient demands, potential enzyme activities (indicating the amount of enzymes) may yield information on the soil nutrient status (Olander & Vitousek, 2000; Sinsabaugh et al., 2008). The low chitinase‐to‐phosphatase‐ratio in the bare soil plots compared to the moss and soil cover plots (Figure 3c) thus indicates a shift from N limitation towards P limitation caused by the moss removal. Peroxidase activity and the ratio of peroxidase to cellobiosidase activity, on the other hand, were enhanced in the bare soil plots (Figure 3e,f), which is most likely not directly related to the high N availability, but an indirect effect of the microbial community change, i.e. increased bacterial abundance, and may partly also be linked to low moisture. Although oxidative enzyme activity is traditionally associated with fungi, the importance of bacteria for oxidative enzyme activity is increasingly recognized (Gittel et al., 2014; Sinsabaugh, 2010), also in connection with increased SOM degradation in soil warming studies (Pold et al., 2015). In contrast to oxidative enzymes, activities of the three hydrolytic enzymes (chitinase, phosphatase and cellobiosidase) were highest in the moss plots, indicating stimulatory effects of the moss layer on microbial production of these enzymes, which may be due to leaching of labile substrates from the moss and the positive influence of mosses on soil moisture.

Generally, the effects of soil cover manipulations on enzyme activities seem to be linked to altered soil physical properties rather than to the removal of the moss litter. Given the slow decomposition rates in the subarctic, the removal of moss litter in the bare soil and artificial soil cover plots had most likely only minor impact on SOM quality at the time of the soil sampling (10 months after the start of the experiment). The rather short time span after the setup of the plots, however, also implies that disturbance effects by the removal of scattered dwarf shrubs in all plots or root trenching may have influenced our results. Increased input of dead fine root biomass and the absence of living roots most likely caused changes in microbial community composition (especially a decline in mycorrhizal abundance) and impacts on soil fauna (Fanin et al., 2019). As these disturbance effects were the same in all plots, this should, however, not have influenced the relative effects of our experimental treatments.

It should be noted in this context that, because of the absence of vascular plants, our experimental plots are not directly comparable to natural plant communities. The objective of our study was to elucidate the mechanisms by which mosses influence soil microbial activity, and these mechanisms can also be assumed to be similarly active in mixed plant communities. However, if mosses were experimentally removed from mixed plant communities, moss removal and the resulting change in N availability would most likely promote growth of vascular plants or alterations in the community composition of vascular plants (Bret‐Harte et al., 2004; Gundale et al., 2012), which would make it difficult to disentangle if impacts on soil microbial activity are directly linked to the absence of mosses or to the functional traits of vascular plants replacing the mosses.

In our experiment we observed that the artificial soil cover plots, which resembled the moss plots in the soil thermal regime, were also similar to the moss plots in soil N availability and some of the microbial process rates (in accordance with our second hypothesis), suggesting a causal link between the effects of mosses on soil N availability and enzyme patterns and their influence on the soil thermal regime. As demonstrated in numerous studies, minor changes in average soil temperature of less than 1°C may significantly affect net N mineralization and inorganic N availability (Marañón‐Jiménez et al., 2019; Rustad et al., 2001; Weedon et al., 2012). The effects of soil cover on the mean soil temperature in summer were, however, not statistically significant, while we observed strong effects on the daily soil temperature fluctuations (Figure 1; Table 1). Although seldom reported, soil temperature amplitude might be as influential on microbial activity as average soil temperature. According to the Arrhenius kinetics of chemical reactions, decomposition rates of SOM theoretically respond exponentially to temperature increase (Davidson & Janssens, 2006), as reported for respiration rates (Carey et al., 2016; Kirschbaum, 2004) and enzyme activities (Fraser et al., 2013; Stone et al., 2012). The high peak soil temperatures in the bare soil plots may thus have contributed more strongly to the acceleration of microbial process rates than a minor change in average soil temperature. Apart from effects during the growing season, soil temperature amplitude may also influence nutrient cycling by affecting the frequency of freeze–thaw events in autumn and spring. Repeated freeze–thaw cycles have been shown to reduce microbial biomass and cause a flush of dissolved N leached from dead microbes (Larsen et al., 2002; Song et al., 2017), but may also amplify potentially mineralizable N by physical disruption of SOM (Steinweg et al., 2008). In our study the number of freeze–thaw events was greatly reduced in the moss plots and fleece‐covered plots compared to the bare soil plots, but intermediate in the plots covered with foam material (Table S2). Although our experiment does not allow to clearly distinguish effects of freeze–thaw events from direct temperature effects on microbial physiology in summer, we conclude that the impacts of the moss and soil cover on N availability and enzyme activities most likely rather reflect effects on soil thermal regime during the growing season than results of freeze–thaw processes in autumn and spring.

Our results demonstrate that mosses not only impact soil N availability via their influence on soil physical properties, but also via sequestration of atmospheric N deposition. Ten months after application of the first N dose, the major part of the added N was still found in the moss layer, and only a small percent of the N was recovered in soil beneath intact moss, compared to around 50% in the bare soil (Table 1). As a consequence of the high N retention by the moss layer, the applied N load of 1.5 g N m^−2^ year^−1^ did not cause any changes in soil N availability, enzyme activities or microbial community composition in the moss plots, which was in accordance with our third hypothesis. Similarly, Gundale et al. (2011) reported that feather mosses prevented effects of N fertilization on soil N concentrations up to a N dose of 1.2 g N m^−2^ year^−1^ in a boreal forest. While these results demonstrate that mosses are efficient in retaining increased atmospheric N loads over time scales of up to several years (Eckstein, 2000; Rousk et al., 2014), indirect effects of increased N deposition on N‐cycling are possible in the longer‐term via changes in decomposition rates of moss biomass and thus altered depth of the moss layer, which in turn affects the insulation capacity of the moss layers (Street et al., 2018).

In contrast to the moss plots, N fertilization increased the availability of dissolved N in the artificial soil cover plots (Figure 2). Interestingly, this was mainly due to an increase in concentrations of native soil N, (as revealed by the analysis of ^15^N, Figure S2), indicating microbial activation by the inorganic N addition. Microbial process data suggest, however, that this was rather an ‘apparent priming effect' (Blagodatskaya & Kuzyakov, 2008) reflecting increased microbial biomass turnover (as also indicated by gross N‐cycling rates) and not a real priming effect of SOM degradation, as we did not observe alterations in enzyme activities by the N addition (Figure 3). In the bare soil plots, on the other hand, N fertilization reduced the concentrations of dissolved native soil N. Together with the lower concentrations of DON, bacterial abundance and phosphatase activities, this suggests that the combination of moss removal and N addition, both causing microbial activation and accelerated N‐cycling, already lead to a depletion of labile substrates in the fertilized bare soil plots. This effect may be comparable with results from long‐term warming studies describing a decline in microbial biomass caused by substrate depletion (Walker et al., 2018).

Although N fertilization affected N pools in the bare soil and artificial soil cover plots, we found surprisingly little alterations in extracellular enzyme activities (Figure 3; Table 2). It is worth noting that the application of an N load of 1.5 g N m^−2^ year^−1^, equaling around ten times the atmospheric background deposition, apparently had less impact on soil enzyme activities than the soil cover treatments. This underlines that the control mosses exert on microbial decomposition processes via their impact on soil physical properties is crucial in the regulation of N cycling in such ecosystems.

A comparison of the applied N load with the N cycling rates in soil shows that, given a gross N mineralization rate of 0.041 g N m^−2^ day^−1^ in the bare soil plots, 1.5 g N/m^2^ were (gross) mineralized by soil microbes in the organic horizon within about 35 days. The slight increase in gross N mineralization rates in the bare soil plots compared to the moss plots would, extrapolated to three summer months, yield an increase in gross ${\text{NH}}_{4}^{+}$ production of 0.9 g N/m^2^ per summer, and a doubling in gross ${\text{NH}}_{4}^{+}$ consumption by the moss removal would cause an increase in gross ${\text{NH}}_{4}^{+}$ consumption of 2.3 g N/m^2^ per summer, which are values at the same order of magnitude as the applied N load with fertilization. This demonstrates the potential strong impact of minor alterations in microbial N dynamics on ecosystem N cycling.

In this study we investigated the effects of bryophytes on soil enzyme activities and N cycling in a subarctic birch forest by a moss removal/artificial soil cover experiment combined with an N fertilization experiment. Our findings highlight the capacity of mosses to reduce soil N availability and retard N cycling. The comparison with artificial soil cover plots suggests that the effect of mosses on N cycling is linked to the thermal insulation capacity of mosses causing low average soil temperature in summer and strongly reduced soil temperature fluctuations, the latter also leading to a decreased frequency of freeze–thaw events in autumn and spring. Our results also showed, however, that the negative temperature effect of mosses on soil microbial activity was in part compensated by stimulatory effects of the moss layer, possibly linked to leaching of labile substrates from the moss. Furthermore, our results reveal that bryophytes efficiently sequester additional N in wet deposition and thus prevent effects of increased atmospheric N deposition on soil N availability and soil processes at the time‐scale investigated in our study. Our study emphasizes the important role of bryophytes in carbon and nutrient cycling in high‐latitude ecosystems and the potential strong impacts of reductions in moss abundance on microbial decomposition processes and nutrient availability in subarctic and boreal forests.

## AUTHORS' CONTRIBUTIONS

M.K. conceived the study, performed the field experiment, analysed samples and data and led the writing of the manuscript; A.M. contributed significantly to all parts of the work and approved the final version of the manuscript.

### PEER REVIEW

The peer review history for this article is available at https://publons.com/publon/10.1111/1365‐2745.13567.

## Supporting information

Supplementary Material

## Data Availability

All data of this study have been archived at Dryad Digital Repository https://doi.org/10.5061/dryad.zcrjdfn95 (Koranda & Michelsen, 2020).
