# Supplementary material for: Mosses reduce soil nitrogen availability in a subarctic birch forest via effects on soil thermal regime and sequestration of deposited nitrogen
Source: J Ecol. 2020 Dec 21;109(3):1424–38. doi: 10.1111/1365-2745.13567 (PMC7986113; doi:10.1111/1365-2745.13567)
Supplement: Supplementary file 1 — Supplementary Material [file JEC-109-1424-s001.docx]

**Supplementary Material**

Table S1

Water holding capacity (WHC) of materials used for the artificial soil cover and of two feather moss species (*Hylocomium splendens* and *Pleurozium schreberi*), which was determined in a pre-experiment. WHC was calculated from the difference in weight between water-saturated state and after 9 days of drying at room temperature. Note that the mosses used for the pre-experiment originated from another site than the study site and were taller than the mosses at the study site (~ 7 cm thickness of the moss cushions compared to ~ 5.5 cm at the study site). Values are unreplicated, as a large range of materials and moss species were tested.

| Moss species / Soil cover material | WHC (L m^-2^) | WHC (g cm^-3^) |
| --- | --- | --- |
| *Hylocomium splendens* (7 cm thickness) | 8.4 | 0.13 |
| *Pleurozium schreberi* (7 cm thickness) | 11.7 | 0.18 |
| Foam material (4 cm thickness) | 6.8 | 0.17 |
| Fleece (4 cm thickness) | 3.1 | 0.08 |

Table S2

Number of freeze-thaw cycles per year at 3 cm soil depth in moss plots, bare soil plots and plots covered with foam material and fleece, respectively (see Method section for details on the calculation). Values are means (n = 3). Significant differences between soil cover treatments are indicated by different letters (p < 0.05).

|  | Moss | Bare soil | Foam | Fleece |
| --- | --- | --- | --- | --- |
| Number of freeze-thaw cycles (no threshold) | 3.7 ^a^ | 16.7 ^b^ | 11.5 ^ab^ | 5.7 ^ab^ |
| Number of freeze-thaw cycles (threshold 0 ± 0.1°C) | 2.0 ^a^ | 12.0 ^b^ | 5.5 ^ab^ | 1.3 ^a^ |

Table S3

Results of linear regression models on the relationship of extracellular enzyme activities and soil temperature and moisture in summer. Analyses were performed with treatment averages (n = 8). Given is R^2^, significance is indicated by ** (p < 0.01), * (p < 0.05) and + (p < 0.1). The direction of significant relationships is indicated by (+) and (-).

|  | Mean soil temperature | Afternoon soil temperature | Soil moisture |
| --- | --- | --- | --- |
| Chitinase | (-) **0.48 ^+^** | (-) **0.79 **** | (+) **0.72 **** |
| Phosphatase | 0.01 | 0.14 | (+) **0.53 *** |
| Cellobiosidase | 0.00 | 0.18 | 0.35 |
| Peroxidase | (+) **0.72 **** | (+) **0.60 *** | 0.24 |


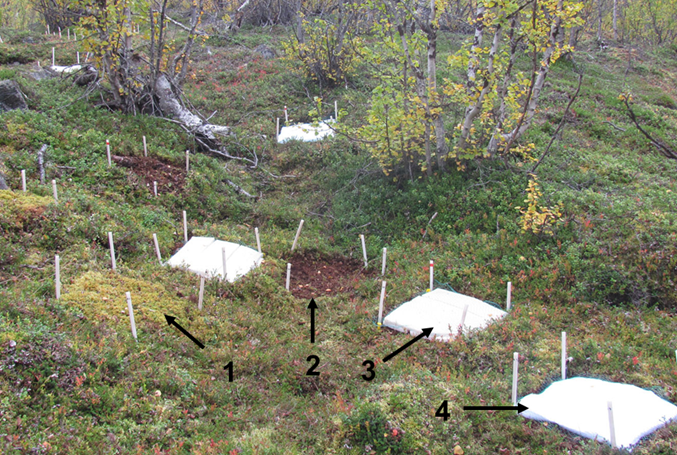


Fig. S1

Field manipulation experiment in an open birch forest close to Abisko (Northern Sweden) consisting of four soil cover treatments: (1) moss, (2) bare soil, (3) foam material (polyurethane), (4) polyester fleece. (*Note that the photo was taken at the start of the experiment. The artificial soil cover got darker in color during the course of the experiment as a result of dust and litter deposition.)*

Fig. S2

Concentration of total dissolved N in water extracts of soil collected in moss plots, bare soil plots and plots covered with foam material and fleece, respectively. In Fig. (a) grey bars represent non-fertilized plots and black bars represent plots fertilized with NH_4_NO_3_. Fig. (b) shows the same values of total dissolved N as in Fig. a, but differentiated into dissolved N originating from native soil N (grey bars) and dissolved N originating from fertilizer N (open bars). Values are means ± SE (n = 6). Significant differences in dissolved native soil N between fertilized and non-fertilized plots of a certain cover treatment (determined by t-test) are indicated by asterisks (*** p < 0.001, ** p < 0.01, * p < 0.05).
